# Core Imaging Library - Part I: a versatile Python framework for tomographic imaging

**DOI:** 10.1098/rsta.2020.0192

**Published:** 2021-08-23

**Authors:** J. S. Jørgensen, E. Ametova, G. Burca, G. Fardell, E. Papoutsellis, E. Pasca, K. Thielemans, M. Turner, R. Warr, W. R. B. Lionheart, P. J. Withers

**Affiliations:** ^1^ Department of Applied Mathematics and Computer Science, Technical University of Denmark, Kongens Lyngby, Denmark; ^2^ Laboratory for Applications of Synchrotron Radiation, Karlsruhe Institute of Technology, Karlsruhe, Germany; ^3^ ISIS Neutron and Muon Source, STFC, UKRI, Rutherford Appleton Laboratory, Didcot, UK; ^4^ Scientific Computing Department, STFC, UKRI, Rutherford Appleton Laboratory, Didcot, UK; ^5^ Henry Royce Institute, Department of Materials, The University of Manchester, Manchester, UK; ^6^ Research IT Services, The University of Manchester, Manchester, UK; ^7^ Department of Mathematics, The University of Manchester, Manchester, UK; ^8^ Institute of Nuclear Medicine and Centre for Medical Image Computing, University College London, London, UK

**Keywords:** computed tomography, X-ray CT, convex optimization, software, image reconstruction

## Abstract

We present the Core Imaging Library (CIL), an open-source Python framework for tomographic imaging with particular emphasis on reconstruction of challenging datasets. Conventional filtered back-projection reconstruction tends to be insufficient for highly noisy, incomplete, non-standard or multi-channel data arising for example in dynamic, spectral and *in situ* tomography. CIL provides an extensive modular optimization framework for prototyping reconstruction methods including sparsity and total variation regularization, as well as tools for loading, preprocessing and visualizing tomographic data. The capabilities of CIL are demonstrated on a synchrotron example dataset and three challenging cases spanning golden-ratio neutron tomography, cone-beam X-ray laminography and positron emission tomography.

This article is part of the theme issue ‘Synergistic tomographic image reconstruction: part 2’.

## 1. Introduction

It is an exciting time for computed tomography (CT): existing imaging techniques are being pushed beyond current limits on resolution, speed and dose, while new ones are being continually developed [1]. Driving forces include higher-intensity X-ray sources and photon-counting detectors enabling respectively fast time-resolved and energy-resolved imaging. *In situ* imaging of evolving processes and unconventional sample geometries such as laterally extended samples are also areas of great interest. Similar trends are seen across other imaging areas, including transmission electron microscopy (TEM), positron emission tomography (PET), magnetic resonance imaging (MRI) and neutron imaging, as well as joint or multi-contrast imaging combining several such modalities.

Critical in CT imaging is the reconstruction step where the raw measured data is computationally combined into reconstructed volume (or higher-dimensional) data sets. Existing reconstruction software such as proprietary programs on commercial scanners are often optimized for conventional, high-quality datasets, relying on filtered back projection (FBP) type reconstruction methods [2]. Noisy, incomplete, non-standard or multi-channel data will generally be poorly supported or not at all.

In recent years, numerous reconstruction methods for new imaging techniques have been developed. In particular, iterative reconstruction methods based on solving suitable optimization problems, such as sparsity and total variation (TV) regularization, have been applied with great success to improve reconstruction quality in challenging cases [3]. This however is highly specialized and time-consuming work that is rarely deployed for routine use. The result is a lack of suitable reconstruction software, severely limiting the full exploitation of new imaging opportunities.

This article presents the Core Imaging Library (CIL)—a versatile open-source Python library for processing and reconstruction of challenging tomographic imaging data. CIL is developed by the Collaborative Computational Project in Tomographic Imaging (CCPi) network and is available from https://www.ccpi.ac.uk/CIL, as well as from [4], with documentation, installation instructions and numerous demos.

Many software libraries for tomographic image processing already exist, such as TomoPy [5], ASTRA [6], TIGRE [7], Savu [8], AIR Tools II [9] and CASToR [10]. Similarly, many Matlab and Python toolboxes exist for specifying and solving optimization problems relevant in imaging, including FOM [11], GlobalBioIm [12], ODL [13], ProxImaL [14] and TFOCS [15].

CIL aims to combine the best of the two worlds of tomography and optimization software in a single easy-to-use, highly modular and configurable Python library. Particular emphasis is on enabling a variety of regularized reconstruction methods within a ‘plug and play’ structure in which different data fidelities, regularizers, constraints and algorithms can be easily selected and combined. The intention is that users will be able to use the existing reconstruction methods provided, or prototype their own, to deal with noisy, incomplete, non-standard and multi-channel tomographic datasets for which conventional FBP type methods and proprietary software fail to produce satisfactory results. In addition to reconstruction, CIL supplies tools for loading, preprocessing, visualizing and exporting data for subsequent analysis and visual exploration. CIL easily connects with other libraries to further combine and expand capabilities; we describe CIL plugins for ASTRA [6], TIGRE [7] and the CCPi-Regularisation (CCPi-RGL) toolkit [16], as well as interoperability with the Synergistic Image Reconstruction Framework (SIRF) [17] enabling PET and MRI reconstruction using CIL.

We envision that in particular two types of researchers might find CIL useful:

— Applied mathematicians and computational scientists can use existing mathematical building blocks and the modular design of CIL to rapidly implement and experiment with new reconstruction algorithms and compare them against existing state-of-the-art methods. They can easily run controlled simulation studies with test phantoms and within the same framework transition into demonstrations on real CT data.
— CT experimentalists will be able to load and pre-process their standard or non-standard datasets and reconstruct them using a range of different state-of-the-art reconstruction algorithms. In this way, they can experiment with, and assess the efficacy of, different methods for compensating for poor data quality or handle novel imaging modalities in relation to whatever specific imaging task they are interested in.

CIL includes a number of standard test images as well as demonstration data and scripts that make it easy for users of both groups to get started using CIL for tomographic imaging. These are described in the CIL documentation and we also highlight that all data and code for the experiments presented here are available as described under Data Accessibility.

This paper describes the core functionality of CIL and demonstrates its capabilities using an illustrative running example, followed by three specialized exemplar case studies. Section 2 gives an overview of CIL and describes the functionality of all the main modules. Section 3 focuses on the optimization module used to specify and solve reconstruction problems. Section 4 presents the three exemplar cases, before a discussion and outlook are provided in §5. Multi-channel functionality (e.g. for dynamic and spectral CT) is presented in the part II paper [18] and a use case of CIL for PET/MR motion compensation is given in [19], both within this same issue; further applications of CIL in hyperspectral X-ray and neutron tomography are presented in [20,21].

## 2. Overview of Core Imaging Library

**Figure 1.**
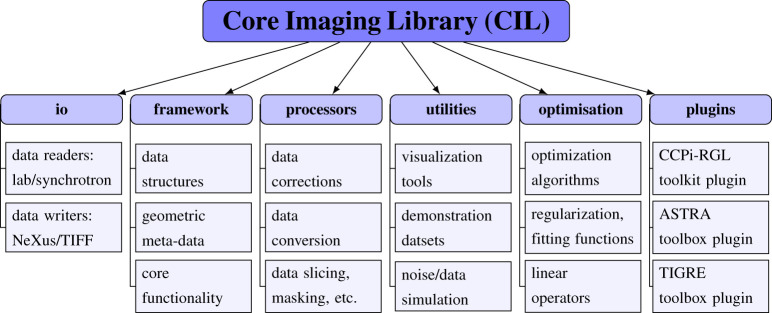
Overview of CIL module structure and contents. The **cil.plugins** module contains wrapper code for other software and third-party libraries that need to be installed separately to be used by CIL.

CIL is developed mainly in Python and binary distribution is currently via Anaconda. Instructions for installation and getting started are available at https://www.ccpi.ac.uk/CIL, as well as from [4]. The present v.21.0 consists of six modules, as shown in figure 1. CIL is open-source software released under the Apache 2.0 license, while individual plugins may have a different license, e.g. **ccpi.plugins.astra** is GPLv3. In the following subsections, the key functionality of each CIL module is explained and demonstrated, apart from **ccpi.optimisation** which is covered in §3.

**Figure 2.**
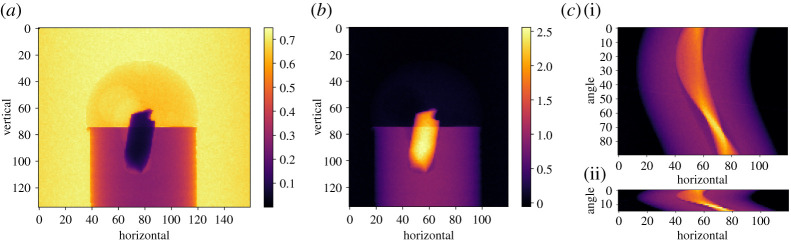
Raw and preprocessed three-dimensional parallel-beam X-ray CT steel-wire dataset. (*a*) Raw transmission projection. (*b*) Scaled, cropped, centred and negative-log transformed projection. (*c*)(i) Sinogram for slice vertical=103, all 90 angles. (*c*)(ii) Same, subsampled to 15 equi-spaced angles.

As a running example (figure 2) we employ a three-dimensional parallel-beam X-ray CT dataset from Beamline I13-2, Diamond Light Source, Harwell, UK. The sample consisted of a 0.5 mm aluminium cylinder with a piece of steel wire embedded in a small drilled hole. A droplet of salt water was placed on top, causing corrosion to form hydrogen bubbles. The dataset, which was part of a fast time-lapse experiment, consists of 91 projections over 180°, originally acquired as size 2560-by-2160 pixels, but provided in [22] downsampled to 160-by-135 pixels.

### (a) Data readers and writers

Tomographic data comes in a variety of different formats depending on the instrument manufacturer or imaging facility. CIL currently supplies a native reader for Nikon’s XTek data format, Zeiss’ TXRM format, the NeXus format [23] if exported by CIL, as well as TIFF stacks. Here ‘native’ means that a CIL AcquisitionData object incl. geometry (as described in the following subsection) will be created by the CIL reader. Other data formats can be read using e.g. DXchange [24] and a CIL AcquisitionData object can be manually constructed. CIL currently provides functionality to export/write data to disk in NeXus format or as a TIFF stack.

The steel-wire dataset is included as an example in CIL. It is in NeXus format and can be loaded using NEXUSDataReader. For example datasets in CIL, we provide a convenience method that saves the user from typing the path to the datafile:

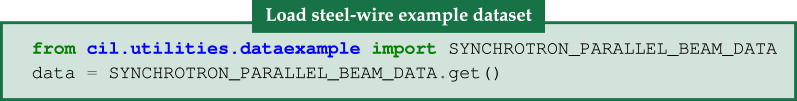


### (b) Data structures, geometry and core functionality

CIL provides two essential classes for data representation, namely AcquisitionData for tomographic data and ImageData for reconstructed (or simulated) volume data. The steel-wire dataset was read in as an AcquisitionData that we can inspect with:

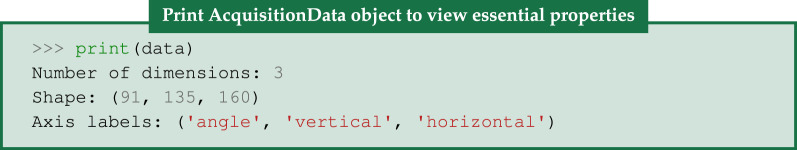


At present, data are stored internally as a NumPy array and may be returned using the method as_array(). AcquisitionData and ImageData use string labels rather than a positional index to represent the dimensions. In the example data, ‘angle’, ‘vertical’ and ‘horizontal’ refer to 91 projections each with vertical size 135 and horizontal size 160. Labels enable the user to access subsets of data without knowing the details of how it is stored underneath. For example, we can extract a single projection using the method get_slice with the label and display it (figure 2*a*) as

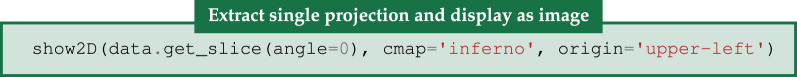

where show2D is a display function in **cil.utilities.display**. show2D displays dimension labels on plot axes as in figure 2; subsequent plots omit these for space reasons.

Both ImageData and AcquisitionData behave much like a NumPy array with support for:

— algebraic operators +, -, etc.,
— relational operators >, >=, etc.,
— common mathematical functions like exp, log and abs, mean, and
— inner product dot and Euclidean norm norm.

This makes it easy to do a range of data processing tasks. For example in figure 2*a*, we note the projection (which is already flat-field normalized) has values around 0.7 in the background, and not 1.0 as in typical well-normalized data. This may lead to reconstruction artefacts. A quick-fix is to scale the image to have background value *ca* 1.0. To do that we extract a row of the data towards the top, compute its mean and use it to normalize the data:

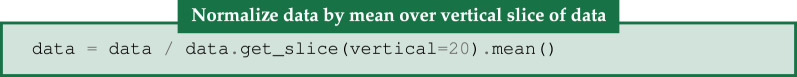

Where possible in-place operations are supported to avoid unnecessary copying of data. For example the Lambert–Beer negative logarithm conversion can be done by:

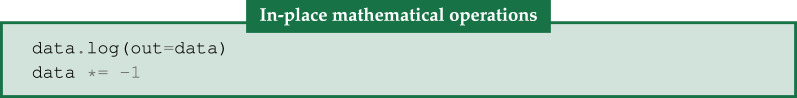


Geometric meta-data such as voxel dimensions and scan configuration is stored in ImageGeometry and AcquisitionGeometry objects available in the attribute geometry of ImageData and AcquisitionData. AcquisitionGeometry will normally be provided as part of an AcquisitionData produced by the CIL reader. It is also possible to manually create AcquisitionGeometry and ImageGeometry from a list of geometric parameters. Had the steel-wire dataset not had geometry information included, we could have set up its geometry with the following call:

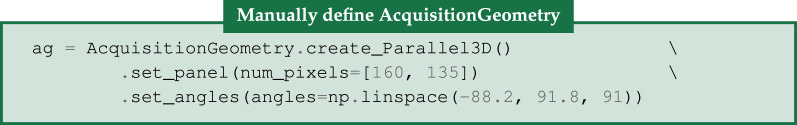

**Figure 9.**
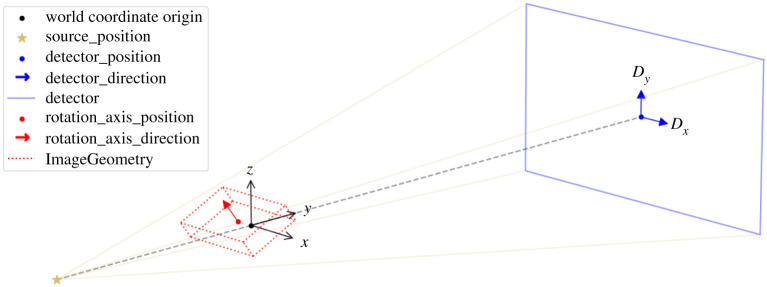
CIL AcquisitionGeometry and ImageGeometry illustrated for the laminography cone-beam setup. Configurable parameters are shown in the legend. Parallel-beam geometry and two-dimensional versions are also available. CIL can illustrate ImageGeometry and AcquisitionGeometry instances as in this figure using show_geometry(ag,ig).

The first line creates a default three-dimensional parallel-beam geometry with a rotation axis perpendicular to the beam propagation direction. The second and third lines specify the detector dimension and the angles at which projections are acquired. Numerous configuration options are available for bespoke geometries; this is illustrated in §4b, see in particular figure 9, for an example of cone-beam laminography. Similarly, ImageGeometry holds the geometric specification of a reconstructed volume, including numbers and sizes of voxels.

### (c) Preprocessing data

In CIL, a Processor is a class that takes an ImageData or AcquisitionData as input, carries out some operations on it and returns an ImageData or AcquisitionData. Example uses include common preprocessing tasks such as resizing (e.g. cropping or binning/downsampling) data, flat-field normalization and correction for centre-of-rotation offset, see table 1 for an overview of Processors currently in CIL.

**Table 1.** Processors currently available in CIL.

name	description
Binner	downsample data in selected dimensions
CentreOfRotationCorrector	find and correct for centre-of-rotation offset
Normaliser	apply flat and dark field correction/normalization
Padder	pad/extend data in selected dimensions
Slicer	extract data at specified indices
Masker	apply binary mask to keep selected data only
MaskGenerator	make binary mask to keep selected data only
RingRemover	remove sinogram stripes to reduce ring artefacts

We will demonstrate centre-of-rotation correction and cropping using Processors. Typically, it is not possible to align the rotation axis perfectly with respect to the detector, and this leads to well-known centre-of-rotation reconstruction artifacts. CIL provides different techniques to estimate and compensate, the simplest being based on cross-correlation on the central slice. First the Processor instance must be created; this is an object instance which holds any parameters specified by the user; here which slice to operate on. Once created the Processor can carry out the processing task by calling it on the targeted dataset. All this can be conveniently achieved in a single code line, as shown in the first line below.

Afterwards, we use a Slicer to remove some of the empty parts of the projections by cropping 20 pixel columns on each side of all projections, while also discarding the final projection which is a mirror image of the first. This produces data90. We can further produce a subsampled dataset data15 by using another Slicer, keeping only every sixth projection.

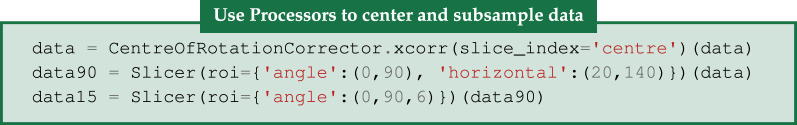


Figure 2 illustrates preprocessing and the final 90- and 15-projection sinograms; mainly the latter will be used in what follows to highlight differences between reconstruction methods.

### (d) Auxiliary tools

This module contains a number of useful tools:

— **dataexample**: Example datasets and test images such as the steel-wire dataset.^1^
— **display**: Tools for displaying data as images, including the show2D used in the previous section and other interactive displaying tools for Jupyter notebooks.
— **noise**: Tools to simulate different kinds of noise, including Gaussian and Poisson.
— **quality_measures**: Mathematical metrics mean-square-error (MSE) and peak-signal-to-noise-ratio (PSNR) to quantify image quality against a ground-truth image.

Some of these tools are demonstrated in other sections of the present paper; for the rest, we refer the reader to the CIL documentation.

### (e) Core Imaging Library plugins and interoperability with Synergistic Image Reconstruction Framework

CIL allows the use of third-party software through *plugins* that wrap the desired functionality. At present, the following three plugins are provided:

— **cil.plugins.ccpi_regularisation**: This plugin wraps a number of regularization methods from the CCPi-RGL toolkit [16] as CIL Functions.
— **cil.plugins.astra**: This plugin provides access to CPU and GPU-accelerated forward and back projectors in ASTRA as well as the FBP and Feldkamp–Davis–Kress (FDK) reconstruction methods for parallel and cone-beam geometries.
— **cil.plugins.tigre**: This plugin currently provides access to GPU-accelerated cone-beam forward and back projectors and the FDK reconstruction method of the TIGRE toolbox.

Furthermore, CIL is developed to be interoperable with the Synergistic Image Reconstruction Framework (SIRF) for PET and MR imaging [17]. This was achieved by synchronizing naming conventions and basic class concepts:

— **sirf**: Data structures and acquisition models of SIRF can be used from CIL without a plugin, in particular with **cil.optimisation** one may specify and solve optimization problems with SIRF data. An example of this using PET data is given in §4c.

We demonstrate here how the **cil.plugins.astra** plugin, or **cil.plugins.tigre** plugin interchangeably, can be used to produce an FBP reconstruction of the steel-wire dataset using its FBP Processor. To compute a reconstruction we must specify the geometry we want for the reconstruction volume; for convenience, a default ImageGeometry can be determined from a given AcquisitionGeometry. The FBP Processor can then be set up and in this instance we specify for it to use GPU-acceleration, and then call it on the dataset to produce a reconstruction:

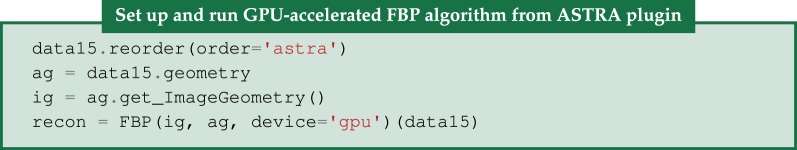

**Figure 3.**
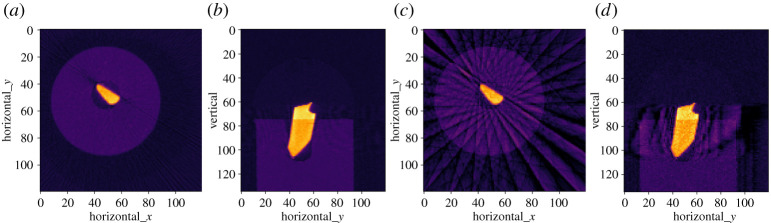
Reconstructions of steel-wire dataset by FBP. (*a*,*b*) Horizontal and vertical slices using 90 projections. (*c*,*d*) Same using 15 projections—showing prominent streak artefacts. Colour range [−0.01, 0.11].

The first line permutes the underlying data array to the specific dimension order required by **cil.plugins.astra**, which may differ from how data is read into CIL. Reconstructions for both the 90- and 15-projection steel-wire datasets are seen in figure 3, with notable streak artefacts in the subsampled case, as is typical with few projections.

## 3. Reconstruction by solving optimization problems

FBP type reconstruction methods have very limited capability to model and address challenging datasets. For example, the type and amount of noise cannot be modelled and prior knowledge such as non-negativity or smoothness cannot be incorporated. A much more flexible class of reconstruction methods arises from expressing the reconstructed image as the solution to an optimization problem combining data and noise models and any prior knowledge.

The CIL optimization module makes it simple to specify a variety of optimization problems for reconstruction and provides a range of optimization algorithms for their solution.

### (a) Operators

The **ccpi.optimisation** module is built around the generic linear inverse problem

3.1 Au=b,

where *A* is a linear operator, *u* is the image to be determined, and *b* is the measured data. In CIL, *u* and *b* are normally represented by ImageData and AcquisitionData, respectively, and *A* by a LinearOperator. The spaces that a LinearOperator maps from and to are represented in attributes domain and range; these should each hold an ImageGeometry or AcquisitionGeometry that match with that of *u* and *b*, respectively.

Reconstruction methods rely on two essential methods of a LinearOperator, namely direct, which evaluates *Av* for a given *v*, and adjoint, which evaluates $A^{*}z$ for a given *z*, where $A^{*}$ is the adjoint operator of *A*. For example, in a LinearOperator representing the discretized Radon transform for tomographic imaging, direct is *forward projection*, i.e. computing the sinogram corresponding to a given image, while adjoint corresponds to *back-projection*.

Table 2 provides an overview of the Operators available in the current version of CIL. It includes imaging models such as BlurringOperator for image deblurring problems and mathematical operators such as IdentityOperator and GradientOperator to act as building blocks for specifying optimization problems. Operators can be combined to create new Operators through addition, scalar multiplication and composition.

**Table 2.** Operators in CIL; and Operators from **cil.plugins.astra** and **cil.plugins.tigre** in bottom two rows.

name	description
BlockOperator	form block (array) operator from multiple operators
BlurringOperator	apply point spread function to blur an image
ChannelwiseOperator	apply the same operator to all channels
DiagonalOperator	form a diagonal operator from image/acquisition data
FiniteDifferenceOperator	apply finite differences in selected dimension
GradientOperator	apply finite difference to multiple/all dimensions
IdentityOperator	apply identity operator, i.e. return input
MaskOperator	from binary input, keep selected entries, mask out rest
SymmetrisedGradientOperator	apply symmetrized gradient, used in TGV
ZeroOperator	operator of all zeroes
ProjectionOperator	tomography forward/back-projection from ASTRA
ProjectionOperator	tomography forward/back-projection from TIGRE

The bottom two rows contain ProjectionOperators from both **cil.plugins.astra** and **cil.plugins.tigre**, which wrap forward and back-projectors from the ASTRA and TIGRE toolboxes, respectively, and can be used interchangeably. A ProjectionOperator can be set up simply by

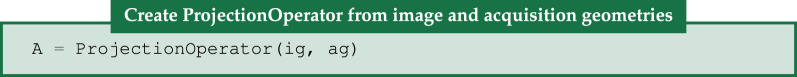

and from the AcquisitionGeometry provided the relevant two-dimensional or three-dimensional, parallel-beam or cone-beam geometry employed; in case of the steel-wire dataset, a three-dimensional parallel-beam geometry.

### (b) Algebraic iterative reconstruction methods

One of the most basic optimization problems for reconstruction is least-squares minimization,

3.2 $$u^{⋆}=\underset{u}{\text{arg min}}||Au-b|{|}_{2}^{2},$$

where we seek to find the image *u* that fits the data best, i.e. in which the norm of the residual *A u* − *b* takes on the smallest possible value; this *u* we denote $u^{⋆}$ and take as our reconstruction.

The conjugate gradient least squares (CGLS) algorithm [25] is an algebraic iterative method that solves exactly this problem. In CIL, it is available as CGLS, which is an example of an Algorithm class. The following code sets up a CGLS algorithm instance—inputs required are an initial image, the operator (here ProjectionOperator from **cil.plugins.astra**), the data and an upper limit on the number of iterations to run—and runs a specified number of iterations with verbose printing:

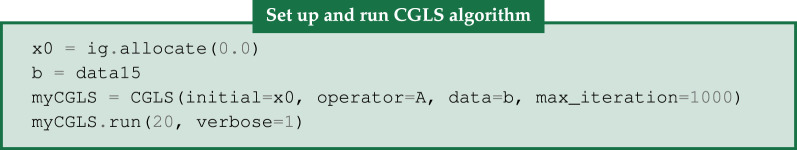

At this point, the reconstruction is available as myCGLS.solution and can be displayed or otherwise analysed. The object-oriented design of Algorithm means that iterating can be resumed from the current state, simply by another myCGLS.run call.

As imaging operators are often ill-conditioned with respect to inversion, small errors and inconsistencies tend to magnify during the solution process, typically rendering the final least squares $u^{⋆}$ useless. CGLS exhibits semi-convergence [26] meaning that in the initial iterations the solution will approach the true underlying solution, but from a certain point the noise will increasingly contaminate the solution. The number of iterations therefore has an important regularizing effect and must be chosen with care.

CIL also provides the simultaneous iterative reconstruction technique (SIRT) as SIRT, which solves a particular weighted least-squares problem [9,27]. As with CGLS, it exhibits semi-convergence, however it tends to require more iterations. An advantage of SIRT is that it admits the specification of convex constraints, such as box constraints (upper and lower bounds) on *u*; this is done using optional input arguments lower and upper:

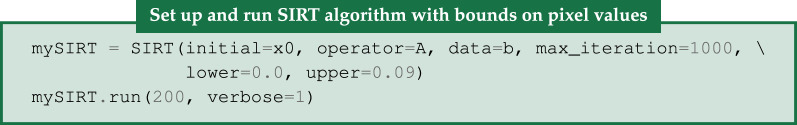

**Figure 4.**
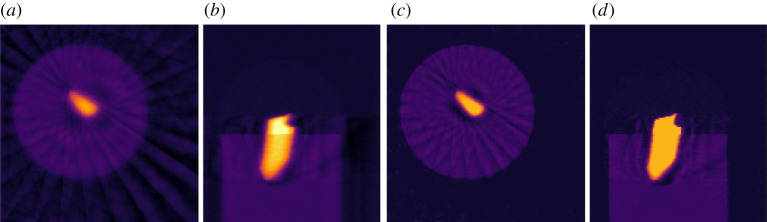
Algebraic iterative reconstruction of 15-projection three-dimensional steel-wire dataset. (*a*,*b*) Horizontal and vertical slices, 20-iteration CGLS reconstruction. (*c*,*d*) Same using SIRT, lower/upper bounds 0.0/0.09. Colour range [−0.01, 0.11].

In figure 4, we see that CGLS reduces streaks but blurs edges. SIRT further reduces streaks and sharpens edges to the background; this is an effect of the non-negativity constraint. In the steel wire example data, the upper bound of 0.09 is attained causing a more uniform appearance with sharper edges.

### (c) Tikhonov regularization with BlockOperator and BlockDataContainer

Algebraic iterative methods like CGLS and SIRT enforce regularization of the solution implicitly by terminating iterations early. A more explicit form of regularization is to include it directly in an optimization formulation. The archetypal such method is Tikhonov regularization which takes the form

3.3 $$u^{⋆}=\underset{u}{\text{arg min}}\{||Au-b|{|}_{2}^{2}+\alpha^{2}||Du|{|}_{2}^{2}\},$$

where *D* is some operator, the properties of which govern the appearance of the solution. In the simplest form *D* can be taken as the identity operator. Another common choice is a discrete gradient implemented as a finite-difference operator. The *regularization parameter* *α* governs the balance between the data fidelity term and the regularization term. Conveniently, Tikhonov regularization can be analytically rewritten as an equivalent least-squares problem, namely

3.4 $$u^{⋆}=\underset{u}{\text{arg min}}||\tilde{A}u-\tilde{b}|{|}_{2}^{2},\;\text{where}\;\tilde{A}=\left(\begin{array}{c} A\\ \alpha D \end{array}\right)\;\text{and}\;\tilde{b}=\left(\begin{array}{c} b\\ 0 \end{array}\right),$$

where the 0 corresponds to the range of *D*. We can use the CGLS algorithm to solve equation (3.4) but we need a way to express the block structure of $\tilde{A}$ and $\tilde{b}$. This is achieved by the BlockOperator and BlockDataContainer of CIL:

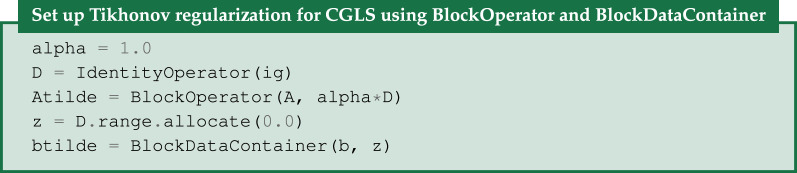

If instead, we want the discrete gradient as *D* we simply replace the second line by:

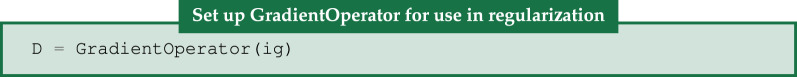

GradientOperator automatically works out from the ImageGeometry ig which dimensions are available and sets up finite differencing in all dimensions. If two or more dimensions are present, D will in fact be a BlockOperator with a finite-differencing block for each dimension. CIL supports nesting of a BlockOperator inside another, so that Tikhonov regularization with a Gradient operator can be conveniently expressed.

**Figure 5.**
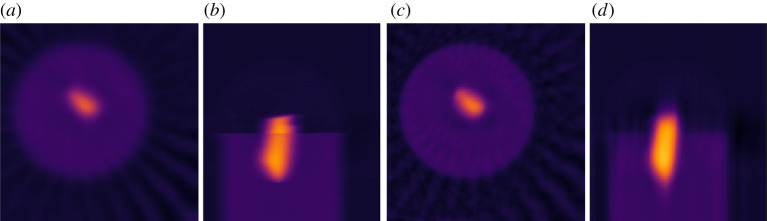
Anisotropic Tikhonov reconstruction of 15-projection three-dimensional steel-wire dataset. (*a*,*b*) Horizontal and vertical slices, Tikhonov regularization with horizontal smoothing (*α*_*x*_ = *α*_*y*_ = 30, *α*_*z*_ = 0.1). (*c*,*d*) Same, with vertical smoothing (*α*_*x*_ = *α*_*y*_ = 0.1, *α*_*z*_ = 60). Colour range [−0.01, 0.11].

In figure 5*a*,*b* Tikhonov regularization with the GradientOperator is demonstrated on the steel-wire sample. Here, *α* governs the solution smoothness similar to how the number of iterations affects CGLS solutions, with large *α* values producing smooth solutions. Here, *α* = 1 is used as a suitable trade-off between noise reduction and smoothing.

The block structure provides the machinery to experiment with different amounts or types of regularization in individual dimensions in a Tikhonov setting. We consider the problem

3.5 $$u^{⋆}=\underset{u}{\text{arg min}}\{||Au-b|{|}_{2}^{2}+\alpha_{x}^{2}||D_{x}u|{|}_{2}^{2}+\alpha_{y}^{2}||D_{y}u|{|}_{2}^{2}+\alpha_{z}^{2}||D_{z}u|{|}_{2}^{2}\},$$

where we have different regularizing operators *D*_*x*_, *D*_*y*_, *D*_*z*_ in each dimension and associated regularization parameters *α*_*x*_, *α*_*y*_, *α*_*z*_. We can write this as the following block least-squares problem which can be solved by CGLS:

3.6 $$u^{⋆}=\underset{u}{\text{arg min}}{\left||\left(\begin{array}{c} A\\ \alpha_{x}D_{x}\\ \alpha_{y}D_{y}\\ \alpha_{z}D_{z} \end{array}\right)u-\left(\begin{array}{c} b\\ 0_{x}\\ 0_{y}\\ 0_{z} \end{array}\right)|\right|}_{2}^{2},$$

where 0_*x*_, 0_*y*_ and 0_*z*_ represent zero vectors of appropriate size.

In figure 5, we show results for *D*_*x*_, *D*_*y*_ and *D*_*z*_ being finite-difference operators in each direction, achieved by the FiniteDifferenceOperator. We show two choices of sets of regularization parameters, namely *α*_*x*_ = *α*_*y*_ = 30, *α*_*z*_ = 0.1 and *α*_*x*_ = *α*_*y*_ = 0.1, *α*_*z*_ = 60. We see in the former case a large amount of smoothing occurs in the horizontal dimensions due to the larger *α*_*x*_ and *α*_*y*_ parameters, and little in the vertical dimension, so horizontal edges are preserved. In the latter case, opposite observations can be made.

Such anisotropic regularization could be useful with objects having a layered or fibrous structure, or if the measurement set-up provides different resolution or noise properties in different dimensions, e.g. for non-standard scan trajectories such as tomosynthesis/ laminography.

### (d) Smooth convex optimization

CIL supports the formulation and solution of more general optimization problems. One problem class supported is unconstrained smooth convex optimization problems,

3.7 $$u^{⋆}=\underset{u}{\text{arg min}}f(u).$$

Here, *f* is a differentiable, convex, so-called *L*-smooth function, that is its gradient ∇f is *L*-Lipschitz continuous: $||\nabla f(u_{1})-\nabla f(u_{2})|{|}_{2}\leq L||u_{1}-u_{2}|{|}_{2},\;\forall \;u_{1},u_{2}$ for some *L* > 0 referred to as the Lipschitz parameter. CIL represents functions by the Function class, which maps an ImageData or AcquisitionData to a real number. Differentiable functions provide the method gradient to allow first-order optimization methods to work; at present CIL provides a Gradient Descent method GD with a constant or back-tracking line search for step size selection. CIL Function supports algebra so the user can formulate for example linear combinations of Function objects and solve with the GD algorithm.

As example we can formulate and solve the Tikhonov problem equation (3.3) with GD as

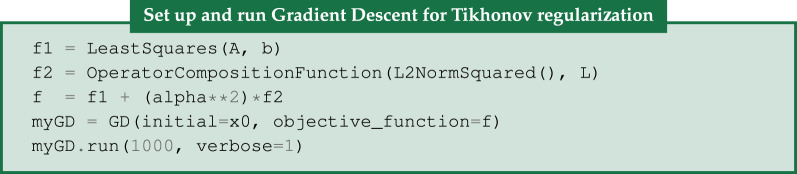


Here, LeastSquares(A,b), representing $||A\cdot -b|{|}_{2}^{2}$, and L2NormSquared, representing $||\cdot |{|}_{2}^{2}$, are examples from the Function class. With OperatorCompositionFunction a function can be composed with an operator, here L, to form a composite function $||L\cdot |{|}_{2}^{2}$. An overview of Function types currently in CIL is provided in table 3. Another example using a smooth approximation of non-smooth TV regularization will be given in §4a.

**Table 3.** Functions in CIL.

name	description
BlockFunction	separable sum of multiple functions
ConstantFunction	function taking the constant value
OperatorCompositionFunction	compose function *f* and operator *A*: *f*(*Ax*)
IndicatorBox	indicator function for box (lower/upper) constraints
KullbackLeibler	Kullback–Leibler divergence data fidelity
L1Norm	*L*^1^-norm: $||x|{|}_{1}=\sum_{i}|x_{i}|$
L2NormSquared	squared *L*^2^-norm: $||x|{|}_{2}^{2}=\sum_{i}x_{i}^{2}$
LeastSquares	least-squares data fidelity: $||Ax-b|{|}_{2}^{2}$
MixedL21Norm	mixed *L*^2,1^-norm: $||(U_{1};U_{2})|{|}_{2,1}=||{\left(U_{1}^{2}+U_{2}^{2}\right)}^{1/2}|{|}_{1}$
SmoothMixedL21Norm	smooth *L*^2,1^-norm: $||(U_{1};U_{2})|{|}_{2,1}^{\text{S}}=||{\left(U_{1}^{2}+U_{2}^{2}+\beta^{2}\right)}^{1/2}|{|}_{1}$
WeightedL2NormSquared	weighted squared *L*^2^-norm: $||x|{|}_{w}^{2}=\sum_{i}(w_{i}\cdot x_{i}^{2})$

### (e) Non-smooth convex optimization with simple proximal mapping

Many useful reconstruction methods are formulated as non-smooth optimization problems. Of specific interest in recent years has been sparsity-exploiting regularization such as the *L*^1^-norm and TV. TV-regularization for example has been shown capable of producing high-quality images from severely undersampled data whereas FBP produces highly noisy, streaky images. A particular problem class of interest can be formulated as

3.8 $$u^{⋆}=\underset{u}{\text{arg min}}\{f(u)+g(u)\},$$

where *f* is *L*-smooth and *g* may be non-smooth. This problem can be solved by the Fast Iterative Shrinkage-Thresholding Algorithm (FISTA) [28,29], which is available in CIL as FISTA. FISTA makes use of *f* being smooth by calling f.gradient and assumes for *g* that the so-called proximal mapping,

3.9 $${\mathrm{prox}}_{\tau g}(u)=\underset{v}{\text{arg min}}\left\{\tau g(v)+\frac{1}{2}||v-u|{|}_{2}^{2}\right\}$$

for a positive parameter *τ* is available as g.proximal. This means that FISTA is useful when *g* is ‘proximable’, i.e. where an analytical expression for the proximal mapping exists, or it can be computed efficiently numerically.

A simple, but useful, case for FISTA is to enforce constraints on the solution, i.e.require *u* ∈ *C*, where *C* is a convex set. In this case, *g* is set to the (convex analysis) indicator function of *C*, i.e.

3.10 $$\iota_{C}(u)=\left\{ \begin{array}{ll} 0 & \text{if}\;u\in C\\ \infty & \text{else}. \end{array}\right.$$

The proximal mapping of an indicator function is simply a projection onto the convex set; for simple lower and upper bound constraints, this is provided in CIL as IndicatorBox. FISTA with non-negativity constraints is achieved with the following lines of code:

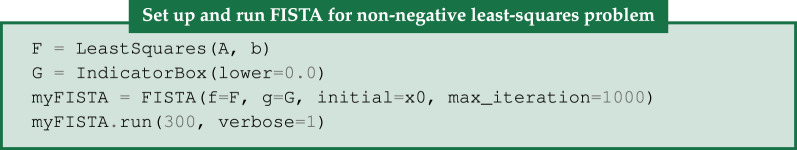

Another simple non-smooth case is *L*^1^-norm regularization, i.e. using $||u|{|}_{1}=\sum_{j}|u_{j}|$ as regularizer. This is non-differentiable at 0 and a closed-form expression for the proximal mapping is known as the so-called soft-thresholding. In CIL, this is available as L1Norm and can be achieved with the same code, only with the second line replaced by

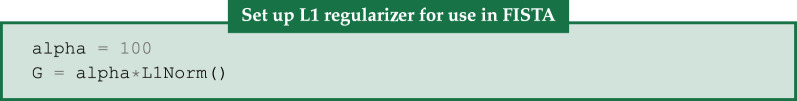

**Figure 6.**
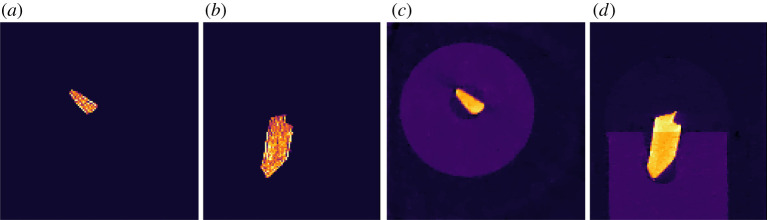
FISTA reconstruction of 15-projection three-dimensional steel-wire dataset. (*a*,*b*) *L*^1^-norm regularization with large regularization parameter of *α* = 30 forces all pixels but in steel wire to zero. (*c*,*d*) TV-regularization with *α* = 0.02 removes streaks and noise and preserves edges. Colour range [−0.01, 0.11].

The resulting steel-wire dataset reconstruction is shown in figure 6.

FISTA can also be used whenever a numerical method is available for the proximal mapping of *g*; one such case is the (discrete, isotropic) TV. TV is the mixed *L*^2,1^-norm of the gradient image,

3.11 $$g_{\text{TV}}(u)=||Du|{|}_{2,1}=||\left(\begin{array}{c} D_{x}\\ D_{y} \end{array}\right)u|{|}_{2,1}={\left||\sqrt{{\left(D_{x}u\right)}^{2}+{\left(D_{y}u\right)}^{2}}|\right|}_{1},$$

where *D* = (*D*_*x*_;*D*_*y*_) is the gradient operator as before and the *L*^2^-norm combines the *x* and *y* differences before the *L*^1^-norm sums over all voxels. CIL implements this in TotalVariation using the FGP method from [29]. Using the FISTA code above, we can achieve this with

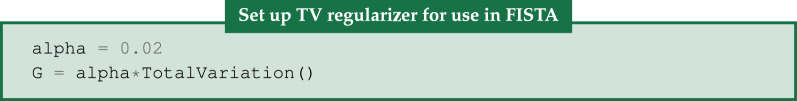

The resulting reconstruction is shown in figure 6 and clearly demonstrates the edge-preserving, noise-reducing and streak-removing capabilities of TV-regularization.

### (f) Non-smooth convex optimization using splitting methods

When the non-smooth function is not proximable, we may consider so-called *splitting methods* for solving a more general class of problems, namely

3.12 $$u^{⋆}=\underset{u}{\text{arg min}}\{f(Ku)+g(u)\},$$

where *f* and *g* are convex (possibly) non-smooth functions and *K* a linear operator. The key change from the FISTA problem is the *splitting* of the complicated *f*(*K*(*u*)), which as a whole may *not* be proximable, into simpler parts *f* and *K* to be handled separately. CIL provides two algorithms for solving this problem, depending on properties of *f* and assuming that *g* is proximable. If *f* is proximable, then the linearized ADMM method [30] can be used; available as LADMM in CIL. If the so-called convex conjugate, $f^{*}$, of *f* is proximable, then the primal dual hybrid gradient (PDHG) method [31–33], also known as the Chambolle–Pock method, may be used; this is known as PDHG in CIL.

In fact, an even wider class of problems can be handled using this formulation, namely

3.13 $$u^{⋆}=\underset{u}{\text{arg min}}\left\{\sum_{i}f_{i}(K_{i}u)+g(u)\right\},$$

i.e. where the composite function *f*(*K* · ) can be written as a separable sum

3.14 $$f(Ku)=\sum_{i}f_{i}(K_{i}u).$$

In CIL, we can express such a function using a BlockOperator, as also used in the Tikhonov example, and a BlockFunction, which essentially holds a list of Function objects.

Here, we demonstrate this setup by using PDHG to solve the TV-regularized least-squares problem. As shown in [33] this problem can be written in the required form as

3.15 $$f=\left(\begin{array}{c} f_{1}\\ f_{2} \end{array}\right)=\left(\begin{array}{c} ||\cdot -b|{|}_{2}^{2}\\ \alpha ||\cdot |{|}_{2,1} \end{array}\right),\;K=\left(\begin{array}{c} A\\ D \end{array}\right),\;g(u)=0.$$

In CIL, this can be written succinctly as (with a specific choice of regularization parameter):

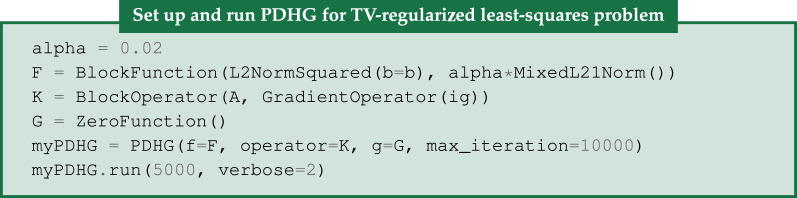


**Figure 7.**
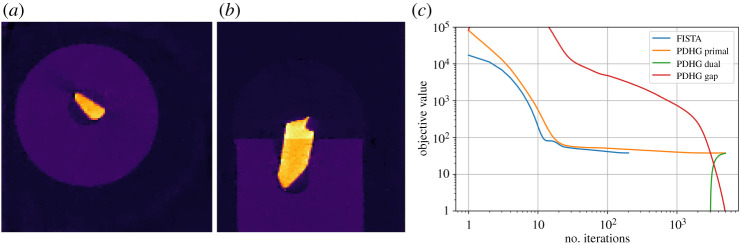
PDHG reconstruction of 15-projection three-dimensional steel-wire dataset. (*a*,*b*) TV-regularization with *α* = 0.02, reproduces the same result as FISTA in figure 6 on the same case and parameter choice, thus validating algorithms against each other. Colour range [−0.01, 0.11]. (*c*) Objective value histories (log-log) for FISTA and PDHG on TV-regularization problem. Both algorithms reach the same (primal) objective value, FISTA taking fewer but slower iterations. The primal-dual gap for PDHG (difference between primal and dual objectives) approaches zero indicating convergence.

Figure 7 shows the resulting steel-wire dataset reconstruction which appears identical to the result of FISTA on the same problem (figure 6), and as such validates the two algorithms against each other.

CIL Algorithms have the option to save the history of objective values so the progress and convergence can be monitored. PDHG is a primal-dual algorithm, which means that the so-called dual maximization problem of equation (3.12), which is referred to as the primal problem, is solved simultaneously. In PDHG, the dual objective values are also available. The primal-dual gap, which is the difference between the primal and dual objective values, is useful for monitoring convergence as it should approach zero when the iterates converge to the solution.

Figure 7*c* compares the primal objective, dual objective and primal-dual gap history with the objective history for FISTA on the same problem. The (primal) objectives settle at roughly the same level, again confirming that the two algorithms achieve essentially the same solution. FISTA used fewer iterations, but each iteration took about 25 times as long as a PDHG iteration. The dual objective is negative until around 3000 iterations, and the primal-dual gap is seen to approach zero, thus confirming convergence. CIL makes such algorithm comparisons straightforward. It should be stressed that the particular convergence behaviour observed for FISTA and PDHG depends on internal algorithm parameters such as step sizes for which default values were used here. The user may experiment with tuning these parameters to obtain faster convergence, for example for PDHG the primal and dual step sizes may be set using the inputs sigma and tau.

In addition to PDHG a stochastic variant SPDHG [34] that can sometimes accelerate reconstruction substantially by working on problem subsets is provided in CIL as SPDHG; this is demonstrated in the Part II article [18] within this issue.

An overview of all the algorithms currently supplied by CIL is provided in table 4.

**Table 4.** Algorithms in CIL.

name	description	problem type solved
CGLS	conjugate gradient least squares	least squares
SIRT	simultaneous iterative reconstruction technique	weighted least squares
GD	gradient descent	smooth
FISTA	fast iterative shrinkage-thresholding algorithm	smooth + non-smooth
LADMM	linearized alternating direction method of multipliers	non-smooth
PDHG	primal dual hybrid gradient	non-smooth
SPDHG	stochastic primal dual hybrid gradient	non-smooth

## 4. Exemplar studies using Core Imaging Library

This section presents three illustrative examples each demonstrating different functionality of CIL. All code and data to reproduce the results are provided, see Data Accessibility.

### (a) Neutron tomography with golden-angle data

This example demonstrates how CIL can handle other imaging modalities than X-ray, a non-standard scan geometry, and easily compare reconstruction algorithms.

Contrary to X-rays, neutrons interact with atomic nuclei rather than electrons that surround them, which yields a different contrast mechanism, e.g. for neutrons hydrogen is highly attenuating while lead is almost transparent. Nevertheless, neutron data can be modelled with the Radon transform and reconstructed with the same techniques as X-ray data.

**Figure 8.**
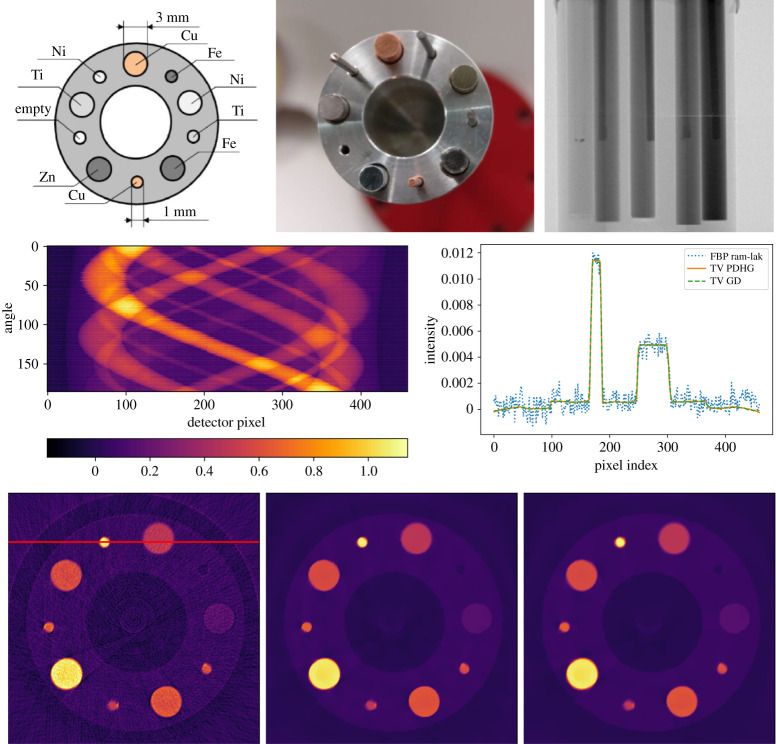
IMAT neutron tomography dataset. Top row: (left) top-view schematic of high-purity elemental metal rod sample; (centre) top-view photograph; (right) single raw projection image showing rods of different absorption. Middle row: (left) preprocessed slice sinogram; (right) horizontal line profile of FBP, PDHG TV and GD TV reconstruction along line shown on image below. Bottom row: (left) slice reconstructions, FBP; (centre) TV reconstruction with PDHG; (right) STV reconstruction with GD. Colour range [−0.002, 0.012].

A benchmarking neutron tomography dataset (figure 8) was acquired at the IMAT beamline [35,36] of the ISIS Neutron and Muon Source, Harwell, UK. The raw data is available at [37] and a processed subset for this paper is available from [38]. The test phantom consisted of an Al cylinder of diameter 22 mm with cylindrical holes holding 1 mm and 3 mm rods of high-purity elemental Cu, Fe, Ni, Ti and Zn rods. 186 projections each 512-by-512 pixels in size 0.055 mm were acquired using the non-standard golden-angle mode [39] (angular steps of $\frac{1}{2}(\sqrt{5}-1)\times 180^{\circ }=111.24\cdots^{\circ }$) rather than sequential small angular increments. This was to provide complete angular coverage in case of early experiment termination and to allow experimenting with reconstruction from a reduced number of projections. An energy-sensitive micro-channel plate (MCP) detector was used [40,41] providing raw data in 2332 energy bins per pixel, which were processed and summed to simulate a conventional white-beam absorption-contrast dataset for the present paper. Reconstruction and analysis of a similar energy-resolved dataset is given in [21].

We use TIFFStackReader to load the data, several Processor instances to preprocess it, and initially FBP to reconstruct it. We compare with TV-regularization, equation (3.11), solved with MixedL21Norm and PDHG using *α* = 1 and 30000 iterations, and further with a smoothed variant of TV (STV) using SmoothMixedL21Norm. The latter makes the optimization problem smooth, so it can be solved using GD, using the same *α* and 10000 iterations.

The sinogram for a single slice is shown in figure 8 along with FBP, TV and STV reconstructions and a horizontal line profile plot as marked by the red line. The FBP reconstruction recovers the main sample features, however it is contaminated by noise, ring artefacts and streak artefacts emanating from the highest-attenuating rods. The TV and STV reconstructions remove these artefacts, while preserving edges. We see that the STV approximates the non-smooth TV very well; this also serves to validate the reconstruction algorithms against one another.

### (b) Non-standard acquisition: X-ray laminography

This example demonstrates how even more general acquisition geometries can be processed using CIL, and how **cil.plugins.ccpi_regularisation** allows CIL to use GPU-accelerated implementations of regularizing functions available in the CCPi-RGL toolkit [16]. Furthermore, unlike the examples up to now, we here employ the ProjectionOperator provided by the TIGRE plugin, though the ASTRA plugin could equally have been used.

Laminography is an imaging technique designed for planar samples in which the rotation axis is tilted relative to the beam direction. Conventional imaging of planar samples often leads to severe limited-angle artefacts due to lack of transmission in-plane, while laminography can provide a more uniform exposure [42]. In TEM, the same technique is known as conical tilt.

An experimental laminography set-up in the so-called rotary configuration was developed [43] for Nikon micro-CT scanners in the Manchester X-ray Imaging Facility. Promising reconstructions of a planar LEGO-brick test phantom were obtained using the CGLS algorithm. Here, we use CIL on the same data [44] to demonstrate how TV-regularization and non-negativity constraints can reduce inherent laminographic reconstruction artefacts. CIL allows the specification of very flexible scan configurations. The cone-beam laminography set-up of the LEGO dataset provides an illustrative case for demonstrating CIL geometry, see figure 9. This particular geometry can be specified as follows, illustrating how different geometry components are used:

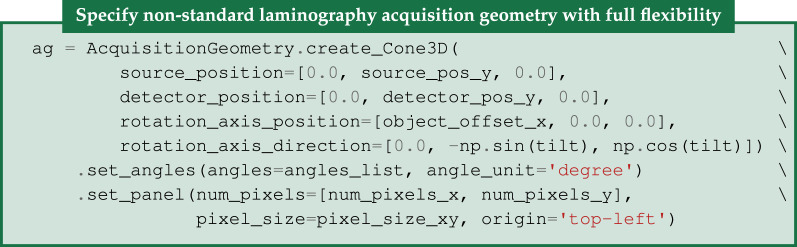


**Figure 10.**
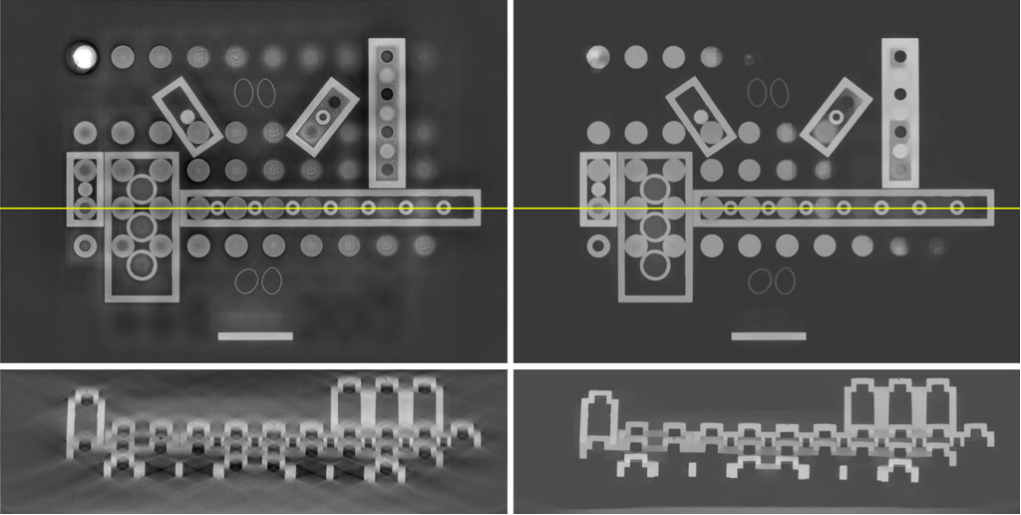
Slices through three-dimensional reconstruction of laminography LEGO sample. Left, top/bottom: LS reconstruction using FISTA, horizontal/vertical slice at yellow line. Right: Same using TVNN, in which laminography artefacts are suppressed while edges are preserved.

The data consist of 2512 projections of 798-by-574 pixels sized 0.508 mm in a 360° cone-beam geometry. We load the data with NikonDataReader and preprocess with a couple of Processor instances to prepare it for reconstruction. For reconstruction we use the GPU-accelerated cone-beam ProjectionOperator from **ccpi.plugin.tigre** and FISTA to solve equation (3.8) for the unregularized least-squares problem (LS) and non-negativity constrained TV-regularized least-squares (TVNN). For TVNN, we use FBP_TV from cil.plugins.ccpi_regularisation which implements a GPU-accelerated version of *g*_TV_, which is faster than, but otherwise equivalent to, using the native CIL TotalVariation. The full three-dimensional volume is reconstructed for LS and TVNN, and figure 10 shows a horizontal and vertical slice through both.

The LEGO bricks are clearly visualized in all reconstructions. The LS reconstruction has a haze in the horizontal slice (top left), which in the vertical slice (bottom left) is seen to amount to smooth directional streaks known to be inherent for laminography; in particular horizontal edges are heavily blurred. On the other hand, fine details in the horizontal plane are preserved, for example the text ‘LEGO’ seen on several knobs to the right.

TVNN (right) reduces the haze and streaks substantially with the LEGO bricks displaying a uniform gray level and the horizontal edges in the vertical slice completely well-defined. However, some fine details are lost, including the ‘LEGO’ text, which is a commonly observed drawback of TV-regularization. Depending on the sample and application, this may or may not be an issue, and if necessary more sophisticated regularizers such as total generalized variation (TGV) could be explored (a CIL example with TGV is given in the Part II article [18]).

As shown, CIL can process very general scan configurations and allows easy experimentation with different reconstruction methods, including using third-party software through plugins.

### (c) PET reconstruction in Core Imaging Library using Synergistic Image Reconstruction Framework

SIRF [17] is an open-source platform for joint reconstruction of PET and MRI data developed by CCP-SyneRBI (formerly CCP-PETMR). CIL and SIRF have been developed with a large degree of interoperability, in particular, data structures are aligned to enable CIL algorithms to work directly on SIRF data. As an example, we demonstrate here reconstruction of the NEMA IQ Phantom [45], which is a standard phantom for testing scanner and reconstruction performance. It consists of a Perspex container with inserts of different-sized spheres, some filled with liquid with higher radioactivity concentration than the background, others with ‘cold’ water (see [45] for more details). This allows assessment of resolution and quantification.

A 60-min PET dataset [46] of the NEMA IQ phantom was acquired on a Siemens Biograph mMR PET/MR scanner at the Institute of Nuclear Medicine, UCLH, London. Due to poor data statistics in PET a Poisson noise model is normally adopted, which leads to using the Kullback–Leibler (KL) divergence as data fidelity. We compare here reconstruction using the ordered subset expectation maximization (OSEM) method [47] available in SIRF without using CIL, and TV-regularized KL divergence minimization using CIL’s PDHG algorithm with a KullbackLeibler data fidelity (KLTV). Instead of a CIL Operator a SIRF AcquisitionModel represents the forward model, and has all necessary methods to allow its use in CIL algorithms.

**Figure 11.**
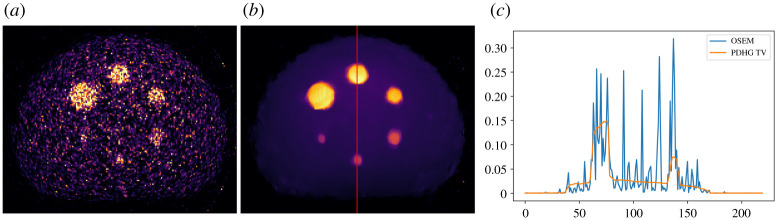
Three-dimensional PET reconstruction of NEMA IQ phantom data using CIL with SIRF data structures. (*a*) OSEM reconstruction (SIRF), horizontal slice. (*b*) KLTV reconstruction (CIL PDHG). Colour range both [0,0.15]. (*c*) OSEM and KLTV profiles along red vertical line on centre plot.

Figure 11 shows horizontal slices through the 220 × 220 × 127-voxel OSEM and KLTV reconstructions and vertical profile plots along the red line. In both cases, the inserts are visible, but OSEM is highly affected by noise. KLTV reduces the noise dramatically, while preserving the insert and outer phantom edges. This may be beneficial in subsequent analysis, however a more detailed comparative study should take post-filtering into account.

The purpose of this example was to give proof of principle of prototyping new reconstruction methods for PET with SIRF, using the generic algorithms of CIL, without needing to implement dedicated new algorithms in SIRF. Another example with SIRF for PET/MR motion compensation employing CIL is given in [19] within this issue.

## 5. Summary and outlook

We have described the CCPi Core Imaging Library, an open-source library, primarily written in Python, for processing tomographic data, with particular emphasis on enabling a variety of regularized reconstruction methods. The structure is highly modular to allow the user to easily prototype and solve new problem formulations that improve reconstructions in cases with incomplete or low-quality data. We have demonstrated the capability and flexibility of CIL across a number of test cases, including parallel-beam, cone-beam, non-standard (laminography) scan geometry, neutron tomography and PET using SIRF data structures in CIL. Further multi-channel cases including temporal/dynamic and spectral tomography are given in [18].

CIL remains under active development with new functionality continually being added, steered by ongoing and future scientific projects. Current plans include:

— adding more algorithms, functions, and operators to support an even greater set of problems, for example allowing convex constraints in smooth problems;
— adding more pre-/postprocessing tools, for example to handle beam hardening;
— adding templates with preselected functions, algorithms, etc. to simplify solving common problems such as TV regularization;
— further integrating with other third-party open-source tomography software through the plugin capability;
— introducing support for nonlinear problems, such as polarimetric neutron spin tomography [48] and electron strain tomography [49]; and
— developing support for multi-modality problems.

CIL is developed as open-source on GitHub, and questions, feature requests and bug reports submitted as issues are welcomed. Alternatively, the developer team can be reached directly at CCPI-DEVEL@jiscmail.ac.uk. CIL is currently distributed through the Anaconda platform; in the future additional modes of distribution such as Docker images may be provided. Installation instructions, documentation and training material is available from https://www.ccpi.ac.uk/CIL, as well as from [4], as are GitHub repositories with source code that may be cloned/forked and built manually. In this way, users may modify and contribute back to CIL.

Finally, we emphasize that a multitude of optimization and regularization methods exist beyond those currently implemented in CIL and demonstrated in the present article. Recent overviews are given for example by [3,50–52] with new problems and methods constantly being devised. CIL offers a modular platform to easily implement and explore such methods numerically as well as apply them directly in large-scale imaging applications.

## Footnotes

1 Available from https://github.com/TomographicImaging/CIL-Data.

## Data accessibility

CIL v21.0 as presented here is available through Anaconda; installation instructions are at https://www.ccpi.ac.uk/CIL. In addition, CIL v21.0 and subsequent releases are archived at [4]. Python scripts to reproduce all results are available from [53]. The steel-wire data set is provided as part of CIL; the original data is at [22]. The neutron data set is available from [38]. The laminography data set is available from [44]. The NEMA IQ PET data set is available from [46].
